# New Approaches to 3D Vision

**DOI:** 10.1098/rstb.2021.0443

**Published:** 2023-01-30

**Authors:** Paul Linton, Michael J. Morgan, Jenny C. A. Read, Dhanraj Vishwanath, Sarah H. Creem-Regehr, Fulvio Domini

**Affiliations:** ^1^ Presidential Scholars in Society and Neuroscience, Center for Science and Society, Columbia University, New York, NY 10027, USA; ^2^ Italian Academy for Advanced Studies in America, Columbia University, New York, NY 10027, USA; ^3^ Visual Inference Lab, Zuckerman Mind Brain Behavior Institute, Columbia University, New York, NY 10027, USA; ^4^ Department of Optometry and Visual Sciences, City, University of London, Northampton Square, London EC1V 0HB, UK; ^5^ Biosciences Institute, Newcastle University, Newcastle upon Tyne, Tyne & Wear NE2 4HH, UK; ^6^ School of Psychology and Neuroscience, University of St Andrews, St Andrews, Fife KY16 9JP, UK; ^7^ Department of Psychology, University of Utah, Salt Lake City, UT 84112, USA; ^8^ Department of Cognitive, Linguistic, and Psychological Sciences, Brown University, Providence, RI 02912-9067, USA

**Keywords:** 3D vision, artificial intelligence, computer vision, navigation, human vision

## Abstract

New approaches to 3D vision are enabling new advances in artificial intelligence and autonomous vehicles, a better understanding of how animals navigate the 3D world, and new insights into human perception in virtual and augmented reality. Whilst traditional approaches to 3D vision in computer vision (SLAM: simultaneous localization and mapping), animal navigation (cognitive maps), and human vision (optimal cue integration) start from the assumption that the aim of 3D vision is to provide an accurate 3D model of the world, the new approaches to 3D vision explored in this issue challenge this assumption. Instead, they investigate the possibility that computer vision, animal navigation, and human vision can rely on partial or distorted models or no model at all. This issue also highlights the implications for artificial intelligence, autonomous vehicles, human perception in virtual and augmented reality, and the treatment of visual disorders, all of which are explored by individual articles.

This article is part of a discussion meeting issue ‘New approaches to 3D vision’.

In November 2021 we held a Royal Society scientific meeting on ‘New approaches to 3D vision’ with the following mission statement:

Leading approaches to computer vision (SLAM: simultaneous localization and mapping), animal navigation (cognitive maps), and human vision (optimal cue integration), start from the assumption that the aim of 3D vision is to produce a metric reconstruction of the environment. Recent advances in machine learning, single-cell recording in animals, virtual reality, and visuomotor control, all challenge this assumption. The purpose of this meeting is to bring these different disciplines together to formulate an alternative approach to 3D vision.

And now was the perfect time to host this meeting. With artificial intelligence's success in 2D vision, attention is now turning to 3D vision. There's been an explosion of interest in 3D image reconstruction (‘A New Trick Lets Artificial Intelligence See in 3D’, *Wired Magazine* [1]), considerable successes in using 3D vision to uncover new biological advances (with DeepMind's AlphaFold [2,3] solving the protein-folding problem), and the suggestion that grounding artificial intelligence in 3D vision will enable better AI (MURI^1^, [4–6]). But 3D vision still remains a challenge for AI [7], and is often regarded as the most difficult question facing robotics and autonomous vehicles [8–10].

At the same time, we are also seeing considerable advances in our understanding of biological vision and navigation. Single-cell recording in freely moving animals has enabled us to understand for the first time how the brain's map of 3D space is organized [11,12], while the emergence of virtual and augmented reality has required that we reconsider the fundamental principles underpinning human 3D vision.

Over 800 people participated in our meeting, with speakers from DeepMind, Google Robotics, Microsoft Research, and Meta (Facebook) Reality Labs, as well as academics from both basic and applied research. Recordings and abstracts of the talks are available on the Royal Society website [13], and links to the talks available in table 1.

**Table 1.** Schedule of talks for the Royal Society meeting ‘New Approaches to 3D Vision’ (1–4 Nov 2021). For links see: https://osf.io/2waby.

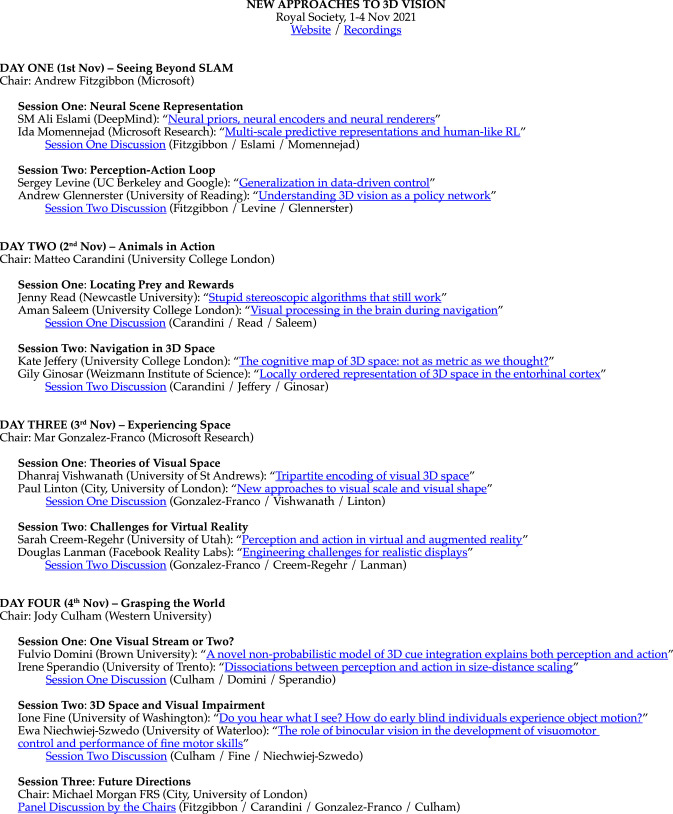

The purpose of our meeting was to capitalize on a brief moment when computer vision, animal navigation, and human vision are all pausing and asking what the most appropriate representation for 3D vision and action really is? On the one hand, it's natural to think that the purpose of 3D vision is to provide us with an accurate model of the environment. On the other hand, in recent years computer vision, animal navigation, and human vision have all been grappling with whether a partial, distorted, or even inconsistent model of the environment might suffice, or perhaps no model at all. And the hope is that by drawing attention to the similarity of these discussions in computer vision, animal navigation, and human vision, we can begin to connect these different approaches, which have evolved relatively independently of one another.

In this Introduction, we outline how these different disciplines have tackled this question and highlight the specific contributions that the papers in this issue make.

## 1. Computer vision

Computer vision originated with 3D vision in the 1960s (Larry Roberts' ‘Machine Perception of Three Dimensional Solids’ (1963) [14–16]). And the emphasis in the 1960s–80s was on using computer vision to reconstruct an accurate 3D model of the environment:

…vision is the *process* of discovering from images what is present in the world, and where it is. (Marr, [17])

The dominant approach to solving this problem was ‘analysis by synthesis’, which treats vision as ‘inverse optics’ or ‘inverse graphics’. For instance, Waltz [18]:

The overall goal of the system is to provide a precise description of a plausible scene which could give rise to a particular [image]…

Horn [19]:

The problem can be viewed as one of inversion: If we understand the projection process which creates images from the three dimensional world, we can hope to reverse this process to recover information about the world.

And Charniak & McDermott [20]:

Given a 2D image, infer the objects that produced it, including their shapes, positions, colors, and sizes.

However, by the early 1980s the pace of progress on 3D world models had slowed ([21], p.ix). And in the mid 1980s and early 1990s, the necessity of an accurate 3D world model began to be questioned on two fronts: Active Vision and Non-Euclidean Geometries.

### (a) Active vision

Advances in computer hardware meant real-time active robotics became a possibility in the mid-1980s. Ruzena Bajcsy at Penn [22,23] and Chris Brown and Dana Ballard at Rochester [24–26] developed robots with active eye movements, complementing previous work by Marty Tenenbaum [27] on computer vision using active lens focusing.

In the mid-1980s, Rodney Brooks [28] developed state-of-the-art autonomous robots by explicitly rejecting the need for a 3D world model [29]:

Internal world models which are complete representations of the external environment, besides being impossible to obtain, are not at all necessary for agents to act in a competent manner

And this was a feature of many Active Vision discussions:

…vision is more readily understood in the context of the visual behaviors that the system is engaged in, and that these behaviors may not require elaborate categorical representations of the 3-D world. (Ballard [25])

The notion of direct coupling of perception and action, without an explicit 3D intermediary, is very appealing. (Blake & Yuille [30], p.173)

Two volumes [30,31] summarize the state of the art up to the early 1990s. And [26] highlights three ways in which Active Vision ‘recast completely the role of vision’, which was adhered to (to varying degrees) by different advocates of Active Vision.

First, active vision is task-specific: ‘an active vision system is far more selfish. It picks out the properties of images which it needs to perform its assigned task, and ignores the rest.’ (Blake & Yuille, [30], p.xv). This was an insight from Yarbus [32]'s work on human eye movements, where patterns of eye movements changed depending on the task.

Second, active vision is dynamic. It only extracts what it needs now. It uses the physical world as its own best model, sampling the physical world when and where it needs to: ‘the visual scene acts as a kind of *external memory buffer* whose unclear parts can be activated by making an eye movement’ (O'Regan & Lévy-Schoen [33]). On this account, ‘the world is its own best model. … The trick is to sense it appropriately and often enough.’ (Brooks [34]).

Third, active vision is adaptive. Since action dictates what visual information is picked-up from the environment, vision is shaped by the organism's interactions with the world, and must be responsive to it. This eradicates a sharp distinction between ‘perception’ and ‘motor control’ modules: ‘there need be no clear distinction between a ‘perception subsystem’, a ‘central system’ and an ‘action system’ (Brooks' [35]). Instead, on Active Vision accounts, modules are tied to specific tasks and behaviours: ‘In the purest form of this model each module incorporates its own perceptual, modelling and planning requirements’. (Brooks [36]).

### (b) Non-Euclidean geometries

In the early 1990s there was also an explosion of interest in using non-Euclidean geometries to solve problems in computer vision:

We usually think of physical space as being embedded in a 3D Euclidean space, in which measurements of length and angles do make sense. It turns out that for artificial systems, such as robots, this is not a mandatory viewpoint and that it is sometimes sufficient to think of physical space as being embedded in an affine or even a projective space. (Faugeras [37])

Affine geometry captures geometry in a loose sense, preserving parallel lines, but not distances or angles. Projective geometry fails to preserve even parallel lines.

First, it was realised that many tasks can be accomplished without a metric model of the environment. There was a sense that ‘computer vision may have been slightly overdoing it in trying at all costs to obtain metric distance information from images.’ (Faugeras [38]). By contrast, ‘Affine structure offers a useful compromise between difficulty of computation and information content.’ (Beardsley *et al.* [39]). Consequently, affine accounts of structure from motion [40], stereo vision [38,41], navigation [39,42,43], and object recognition [44,45] soon emerged.

Second, even when metric scene estimates are necessary, they can be achieved more easily and directly simply by adding constraints to affine geometry, rather than attempting full 3D scene reconstruction: ‘one can estimate all 3D invariants of the scene directly from the images, *without* performing an explicit 3D reconstruction of the scene.’ (Faugeras [37]).

### (c) LIDAR

By contrast, the early 2000s saw a strong re-emergence of Euclidean 3D maps in computer vision. Two developments, LIDAR and SLAM, were key to this re-emergence.

**Figure 1.**
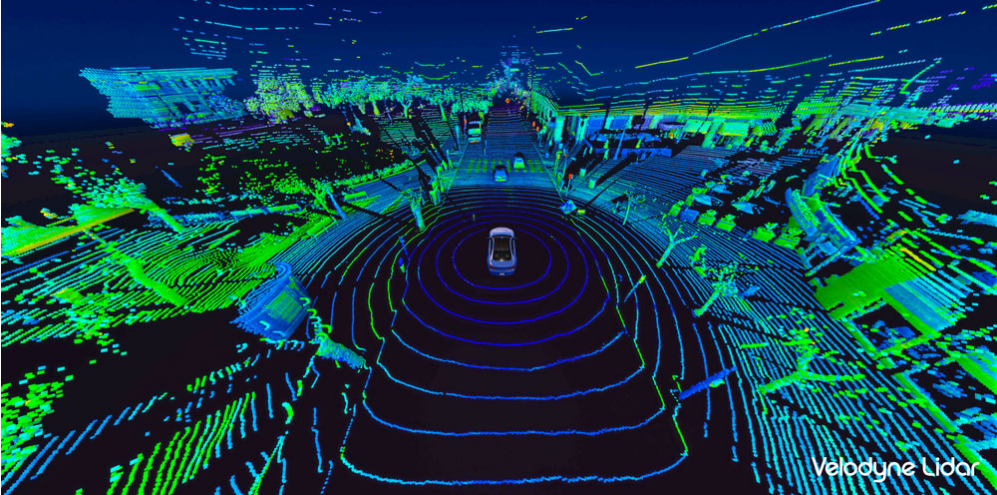
The 360° point cloud created by the Alpha Prime Velodyne Lidar. © Velodyne Lidar. (Online version in colour.)

LIDAR (light detection and ranging) was invented in the 1960s [46]. It estimates the distance of an object in a certain direction by emitting a laser pulse and timing how long it takes to return. LIDAR became synonymous with computer vision in 2005 when ‘Stanley’ (a LIDAR-equipped car) won the 2nd self-driving car DARPA Grand Challenge [47] (previously, all cars had failed the 1st DARPA Grand Challenge). Velodyne's 360° LIDAR was also launched during the 2nd DARPA Grand Challenge, producing a high resolution 360° map of the environment (figure 1). This soon became the industry standard, used by 5 of the 6 finishers of the 3rd DARPA Grand Challenge in 2007 [48,49] as well as Google's self-driving car project (Waymo) [49].

However, the success of deep learning since 2012 [50] has challenged the importance of LIDAR for self-driving cars, leading to a split in the industry. While LIDAR remains a key feature of most self-driving car projects (Waymo (Google), Baidu, Cruise (General Motors)), LIDAR has been explicitly rejected by Tesla since 2013, when its self-driving car program began [51]. Indeed, in 2021 Tesla removed the only range-finding component (radar) from their self-driving cars, with a rationale that explicitly evokes human vision: ‘Humans drive with eyes & biological neural nets, so makes sense that cameras & silicon neural nets are only way to achieve generalized solution to self-driving.’ [52].

### (d) SLAM

SLAM (simultaneous localization and mapping) is the ability of a robot to build a map of its environment, whilst locating itself relative to this map as it navigates. As Thrun *et al.* [53] noted in 2000:

Building maps when a robot's locations are known is relatively straight-forward … Conversely, localizing a robot when a map is readily available is also relatively well understood … In combination, however, the problem is hard.

SLAM remained an intractable problem until the early 2000s. As Durrant-Whyte and colleagues [54] wrote in 1996:

The solution to the simultaneous localisation and map building (SLAM) problem is, in many respects a ‘Holy Grail’ of autonomous vehicle navigation research.

But by 2006, Durrant-Whyte & Bailey [55] could reasonably claim that:

The ‘solution’ of the SLAM problem has been one of the notable successes of the robotics community over the past decade.

So, what changed between 1996 and 2006?

First, the emergence of ‘Probabilistic Robotics' [56] led to a number of Bayesian solutions to the SLAM problem. The first, and by far the most influential, was Extended Kalman Filter (EKF) SLAM (e.g. [57]), derived from Smith *et al.* [58]'s concept of a ‘stochastic map’:

…rather than treat spatial uncertainty as a side issue in geometrical reasoning, we believe it must be treated as an intrinsic part of spatial representations. In this paper, spatial uncertainty will be tied together in a representation called the *stochastic map*. It contains estimates of all the spatial relationships, their uncertainties, and their inter-dependencies.

Another notable solution was FastSLAM [59,60].

Second, early attempts at SLAM combined odometry (motion sensors) with range-finders like sonar [61], radar [62], or lidar [63]. In the early 2000s vision-based systems (Visual SLAM) began to supersede these approaches. Davison & Murray combined SLAM with Active Vision to enable autonomous navigation for a robot with an active stereo head [64–66]. And with MonoSLAM, Davison and colleagues went one step further, using structure from motion to enable SLAM with a single moving camera with no motion sensors or motor commands [67,68].

However, SLAM has two notable shortcomings. First, SLAM is unable to build-up an intuitive understanding of the environment (Gupta *et al.* [69]): ‘These maps are built purely geometrically, and nothing is known until it has been explicitly observed, even when there are obvious patterns.’ New approaches therefore seek to augment SLAM with deep learning [70–74] (see also 3D semantic scene graphs [75–77]). Others seek an alternative to SLAM in deep reinforcement learning [69,78–83] or deep learning [84].

Second, and more fundamentally, SLAM is biologically implausible:

…humans can effectively navigate small and large environments and yet are unlikely to build internally large-scale metric reconstructions of spaces akin to traditional SLAM systems… (Henriques & Vedaldi [84])

Look at the inner workings of most map building algorithms … and there is a strong likelihood of finding Cartesian (*x*, *y*, *z*) representations of the locations of features. It is not clear this is the best way to do things on such a low level, or that biological brains have any similar representation… (Davison [66], p.1)

Especially when SLAM is given an explicit Extended Kalman Filter articulation:

It certainly does not seem that we store the equivalent of a huge covariance matrix relating to the uncertainties in our estimates of the positions of all the features in a certain area. (Davison [66])

This suggests that the extensive metric reconstruction of the environment proposed by SLAM is not necessary for vision or navigation, and is therefore unnecessarily complicated.

### (e) Reinforcement learning

**Figure 2.**
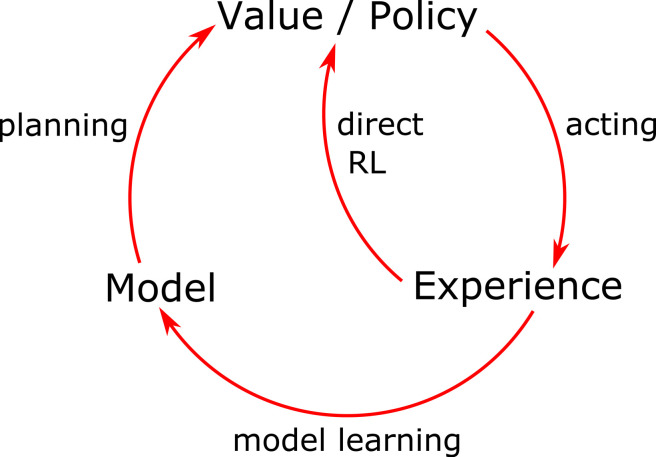
Diagram of Dyna-Q [85] redrawn from [86], p.7, which incorporates both ‘model-based’ (‘value/policy’ → ‘experience’ → ‘model’ → ‘value/policy’) and ‘model-free’ (‘value/policy’ → ‘experience’ → ‘value/policy‘) reinforcement learning. (Online version in colour.)

An alternative approach is reinforcement learning. Reinforcement learning is the process of learning which action to take to maximize the rewards in a given context. Reinforcement learning can take two forms: model-based and model-free, as summarized in figure 2.

Model-based reinforcement learning learns a model of the world in order to predict the rewards of potential actions. By contrast, model-free reinforcement learning learns a direct mapping from the inputs to the actions that maximize rewards.

The earliest work on reinforcement learning was model-based. Richard Sutton and Andrew Barto saw themselves as building ‘an adaptive network that constructs and uses an internal model of its world’ [87], as envisioned by Kenneth Craik:

If the organism carries a ‘small scale model’ of external reality and of its own possible actions within its head, it is able to try out various alternatives, conclude which is the best for them, react to future situations before they arise… ([88] quoted in [87])

By contrast, in the late 1980s ‘model-free’ approaches (such as ‘temporal difference’ [89] and ‘Q-learning’ [90,91]), which do away with a world model altogether, came to the fore. Model-free approaches are:

explicitly trial-and-error learners – viewed as almost the *opposite* of planning. (Sutton & Barto [86], p.7)

And Sutton & Barto [86], p.12 explain the advantages of this approach:

Because models have to be reasonably accurate to be useful, model-free methods can have advantages over more complex methods when the real bottleneck in solving a problem is the difficulty of constructing a sufficiently accurate environment model.

Reinforcement learning came to the public consciousness in 2015 when DeepMind's Deep Q-Network (DQN) achieved human-level performance in Atari computer games [92], and in 2016 when their AlphaGo defeated the world's top Go player [93]. Both relied on ‘deep’ model-free reinforcement learning, where the mapping from inputs to actions is learned by a deep (multilayer) neural network. (Earlier notable success, such as IBM's TD-Gammon [94], which performed competitively at the 1992 World Cup of Backgammon, relied on a ‘shallow’ (single layer) neural network.)

Model-free reinforcement learning challenges the traditional relationship between perception, planning, and action. When DeepMind's Deep Q-Network ‘plays’ Space Invaders, all it learns is the mapping between the pixels on the screen (input) and the buttons to press (output). This is why it's described as a ‘pixels to action’ approach [95]. And end-to-end pixel-to-action training has been successfully applied to robot object manipulation [96], navigation [97–100], driving [101,102], and 3D video games [103].

At our meeting, Sergey Levine [104] argued that this pixel-to-action approach improves performance since robots learn to extract the most relevant representations for the task from the visual input. Similarly, Zhou *et al.* [105] note in ‘Does computer vision matter for action?’

These models bypass explicit computer vision entirely. They do not incorporate modules that perform recognition, depth estimation, optical flow, or other explicit vision tasks. The underlying assumption is that perceptual capabilities will arise in the model as needed, as a result of training for specific motor tasks. This is a compelling hypothesis that, if taken at face value, appears to obsolete much computer vision research.

But the rise of this pixel-to-action approach raises two fundamental questions.

First, would visuomotor control benefit from having an explicit 3D depth map? On the one hand, [105] found that having an explicit depth map and scene segmentation for input significantly improved performance in certain tasks and, even if it didn't (e.g. urban driving), it helped the model to generalize to new and unseen environments (see also [106–111]). And [69,78] also argue that navigation is improved if agents are able to build a top-down Euclidean map of the scene on which to plan their strategies. On the other hand, in this issue Levine & Shah [104] argue that what matters for navigation is traversability, not 3D geometry: tall grass is traversable, even though it looks like a barrier, whilst mud is not traversable, even though it looks like a flat surface, and an explicit representation of 3D geometry is an unnecessary bottleneck to learning traversability. Finally, a third alternative is to use a depth map, but to learn the depth map as part of reinforcement learning, rather than as an input to reinforcement learning [98].

Second, even if this pixel-to-action approach doesn't have an explicit 3D map of the environment, does it effectively learn an implicit 3D map? As Zhu *et al.* [97] note: ‘Our method is considered *map-less*. However, it possesses implicit knowledge of the environment.’ What is the nature of this implicit knowledge? This question has sparked collaborations between computer vision and psychology. On the one hand, the Glennerster & Torr labs study the specific spatial representation in Zhu *et al.* [97], and find ‘only a weak correlation between distance in the embedding space and physical distance between observable locations' [112]. By contrast, SoftBank Robotics and Kevin O'Regan explore the possibility that a Euclidean representation of space emerges organically from reinforcement learning [113–116].

### (f) Deep learning

The Summer Vision Project (1966) [117] defined the mission statement for computer vision as moving from 2D pixels → 3D surfaces → object recognition. But when deep neural networks finally achieved human levels of performance in object recognition, they did this by skipping the 3D surfaces step and going straight from 2D pixels → object recognition [50]. Contrast this with the Summer Vision Project (1966) [117] which assumed: ‘It will be impossible to do this without considerable analysis of shape and surface properties'.

Indeed, 3D vision appeared something of a sticking point for neural networks. Henriques & Vedaldi [84] summarized the position in 2018:

Despite these successes [in 2D images], … several aspects of image understanding remain difficult to approach directly using deep distributed representations. One of them is reasoning about 3D space and geometry…

**Figure 3.**
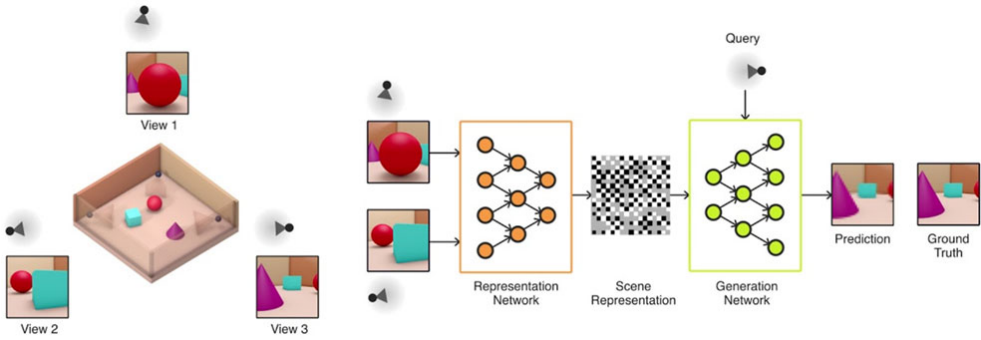
DeepMind's ‘neural scene representation and rendering’ [106]. First, the ‘Representation Network’ creates a low-dimensional ‘Scene Representation’ based on images from a number of different views (View 1 and View 2). Second, the ‘Generation Network’ uses this low-dimensional ‘Scene Representation’ to predict what View 3 will be. © authors. (Online version in colour.)

So, can we teach deep neural networks to reason about 3D space? Here we have to be careful to distinguish neural networks that reason about 3D space from neural networks *that merely act as if they do*. Two general approaches have emerged:

1. Geometric deep learning (Explicit 3D model): Neural networks can be trained to do traditional 3D computer vision. For instance, take a single 2D image as an input and output an explicit 3D model of the object, either as voxels [118–120], point clouds [121], or meshes [122,123]. But it's unclear whether these neural networks are actually reasoning in 3D space (performing a spatial reconstruction of the scene) or merely performing object recognition (searching for a matching 3D template). In ‘What Do Single-view 3D Reconstruction Networks Learn’ [124], three of the then leading models [125–127] are tested, with the conclusion that the ‘current state of the art in single-view object reconstruction does not actually perform reconstruction but image classification.’ Indeed, for many the blurring of 3D reconstruction and object recognition is intentional [118–120,128–130].

2. Neural scene representations (Implicit 3D model): A different approach is to have neural networks learn 3D spatial layout implicitly rather than explicitly. At our meeting, Ali Eslami presented DeepMind's landmark work on ‘neural scene representation’ [106,131] (figure 3). Their network is provided with 2D images of a scene, but is never explicitly told to build a 3D model. Instead, DeepMind reason that if their network can take 2D images, encode a low-dimensional description of the scene, and then use this low-dimensional description to imagine what the scene would look like from a new viewpoint, then the network must have implicitly learnt the 3D layout of the scene. Contrast this with models that just try to predict how 2D pixels on a screen will change with a specific action, without trying to build an intermediate 3D representation [132].

However, since the ‘scene representation’ (low-dimensional description) is not interpretable by humans (it's not an explicit 3D model), we have to do experiments to indirectly reveal the nature of the representation. First, Eslami *et al.* [106] show that the same representation can be used to produce a top-down map of the scene, supporting the idea of an implicit 3D representation. However, they also note that changing the 2D input view of the scene changes the representation, even though it shouldn't if the network were learning a truly view-invariant representation of 3D space. Second, the tension between reconstruction and recognition re-emerges, with Tung *et al.* [73] arguing that failure cases in Eslami *et al.* [106] demonstrate that their ‘geometry-unaware models may be merely memorizing views with small interpolation capabilities, as opposed to learning to spatially reason.’

Both [73] and [133] use failure cases in Eslami *et al.* [106] to argue that learning in explicit 3D coordinates is necessary for true 3D understanding. Consequently, the current literature is primarily focused on ‘neural fields’ [134], ‘neural scene representations' that learn in explicit 3D coordinates.

What's interesting is that these models are implicit in a different sense. The scene representation isn't an output (an interpretable or non-interpretable model) that the network produces, but the neural network itself. So you input a point in 3D space, and the network outputs some property of that point in 3D space, for instance occupancy [135], distance [136], colour + distance [133] or colour + occupancy [137]. From this, the network implicitly learns a continuous (and uninterpretable) mathematical formula that approximates the scene.

**Figure 4.**
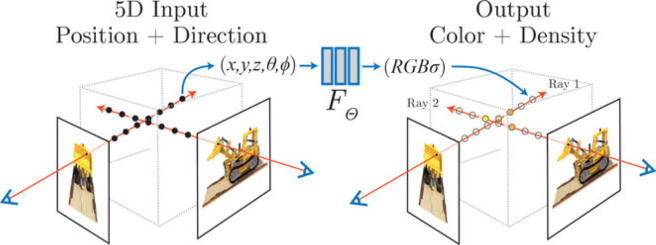
‘Neural Radiance Fields' (NeRFs) [138]. NeRFs generate images by sampling points along a ray (5D input = position + direction) and the network (*F_Θ_*) outputs a colour and density value for each sampled point. NeRF is ‘geometry aware’ since its inputs are explicitly in 3D coordinates (*x,y,z*). © authors. (Online version in colour.)

Interest in these approaches exploded in 2020 with ‘Neural Radiance Fields' (NeRFs) [138] (figure 4), a collaboration between Google, Berkeley and UC San Diego, which takes as inputs the location of a point in 3D space and the direction it's being viewed, and outputs the colour and density of the point in 3D space. This means it can capture the reflective properties of the surfaces, producing photorealistic 3D renderings of real scenes.

However, two challenges remain:

First, like SLAM, ‘Neural Radiance Fields’ (NeRFs) are purely geometric. They can't use prior knowledge about the common structure of scenes to make common sense inferences. Instead, they rely purely on multi-view consistency, which can be achieved without deep learning [139]. This means the original NeRF paper required around 20–60 images per scene [138]. By contrast, recent deep learning approaches such as ‘pixelNeRF’ [140] and ‘NeRF-VAE’ [141] learn common image features across scenes, enabling new scenes to be reconstructed with just 2–3 images.

Second, NeRFs are biologically implausible. They work by giving a colour and density to each point in 3D space. And, to render a new image, they have to ‘ray march’ (by integrating the colours and densities of each point in 3D space that lie along the ray corresponding to each pixel (figure 4)). But this can't be what the human brain is doing. Instead, Vincent Sitzmann and Josh Tenenbaum [1] advance their ‘light field network’ [142] as a more biologically plausible approach because it estimates the colour of each ray (rather than each point along a ray), and builds on earlier work in human vision that employs light-field concepts (‘plenoptic function’ [143], ‘optic array’ [144]).

## 2. Animal navigation

Studying animals with very different eyes and brains from our own opens up new ways of thinking about vision and navigation.

First, the different optics of animal eyes may lead to different depth cues being prioritised. For instance, jumping spiders [145,146], squid [147], and even the prehistoric trilobite [148] have been shown to rely heavily on defocus blur [149], whilst chameleons appear to rely on accommodation (the ability to change the focus of the eye) [150].

Second, if animals have overlapping eyes, how they compute depth from binocular disparity (the difference in the images projected to the two eyes) may be very different [151–153]. In this issue we contrast humans and insects. Michael Morgan [154] explores the complexity of disparity processing in humans. By contrast, Jenny Read [155] explores how an animal with more limited computational capacities, such as the praying mantis, could extract distance information from disparity without the complexities of human vision (such as matching the points in the two eyes or extracting a depth map). Other studies of stereo vision in animals include owls [156], toads [157], and cuttlefish [158].

Third, how different animals navigate the 3D world may be very different as well. Rodney Brooks explains how insects inspired his rejection of 3D models in robotics: ‘Look at an insect, it can fly around and navigate with just a hundred thousand neurons. It can't be doing this very complex symbolic mathematical computations. There must be something different going on.’ [159]. See also work by Barbara Webb [160–163] as well as [164]. Recent work at the intersection of insect navigation, computation, and robotics includes work on bees [165,166], flies (Fly-Net: [167]), and ants [168–170].

### (a) Cognitive maps

However, the key paradigm for animal navigation over the past 120 years has been rodent navigation in mazes. The hope is that it will teach us about mammal navigation in general, and potentially provide important insights about how humans navigate the world. Navigation had its ‘cognitive revolution’ a decade before the rest of psychology when Tolman [171] rejected behaviourism, arguing that the relationship between the stimulus (maze) and the rat's response was mediated by the rat constructing a ‘cognitive map’ of the maze. This insight appeared to be confirmed by two findings that ultimately won the Nobel Prize in 2014.

**Figure 5.**
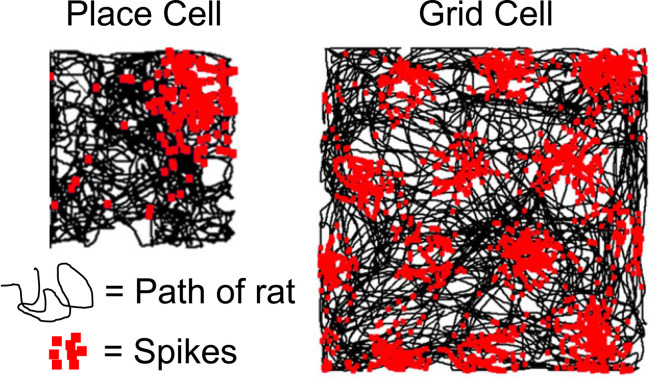
A place cell's firing field (left) and a grid cell's grid of firing fields (right) as a rat moves around an enclosure. Recorded by Elizabeth Marozzi. From [172]. © authors. (Online version in colour.)

First, in the early 1970s ‘place cells’ were found in the hippocampus. These cells fire when an animal is in a specific place in the environment (figure 5) [173]. In *The hippocampus as a cognitive map* [174,175], O'Keefe & Nadel argued that the hippocampus functioned as Tolman's cognitive map. Going beyond Tolman, they argue that this cognitive map had four properties. First, it is ‘Euclidean’ (‘the metric of the cognitive map is Euclidean’ [174]). Second, it is ‘absolute’ (it organizes or structures our experience: ‘the brain must come equipped to impose a 3D Euclidean framework on experience’ [175]). Third, it is ‘world-centred’ or ‘allocentric’ (‘a non-centred stationary framework through which the organism and its egocentric spaces move’ [174]. And fourth, it is ‘innate’ (‘this framework is part of the innate machinery of the organism’ [174]). Whether this ‘Euclidean’ ideal is borne out by the subsequent data is the key concern of this section.

Second, in the mid-2000s ‘grid cells’ were found in the entorhinal cortex. These cells fire when an animal crosses one of the cell's ‘firing fields’: locations in space arranged in a hexagonal grid that cover the entire area (figure 5) [176,177]. Each grid cell's hexagonal grid has its own scale (spacing between the firing fields), orientation (rotation of the firing fields), and phase (grid shifted in a certain direction). When grid cells were found, it was immediately hypothesized that their regular geometry could provide a metric map of the environment [178]: a map that preserves both the angles and distances between points, enabling path integration (for distance estimation) and vector navigation (for shortcuts) [179]. As Grieves & Jeffery [180] note: ‘Given their mathematical and geometric properties it is almost hard to believe these cells exist at all’.

### (b) Distortions in place cells and grid cells

However, the spatial mappings of both place cells and grid cells are subject to significant distortions [181,182], challenging the notion that they provide a metric map of the environment.

First, grid cells are loosely anchored to the environment, with a grid cell's firing fields being stable across time. But what this means is that if the environment is artificially expanded or contracted, the grid cell's firing fields will also be expanded or contracted by roughly 50%, creating a distorted geometry and metric to the space [183,184]. Similar distortions are also seen in place cells [185].

Second, grid cell firing fields are distorted by the borders of the environment [186], and there's evidence that these distortions impact spatial memory [187]. For instance, in a square environment these distortions lead to an elliptical grid [188], whilst in an irregular environment, like a trapezoid, the grid is almost obliterated [189,190]. As Krupic *et al.* [189] conclude:

These results challenge the idea that the grid cells system can act as a universal spatial metric for the cognitive map as grid patterns change markedly between enclosures and even within the same enclosure.

Again, similar distortions are also seen in place cells [190].

Third, Boccara *et al.* [191] found that rewards also distort the regular grid arrangement of grid cell firing fields:

Many grid fields moved toward goal locations, leading to long-lasting deformations of the entorhinal map.

Put simply, the entorhinal cortex (grid cell) cognitive map is attracted to goals: ‘This demonstrates the influence of nongeometrical cognitive factors on the grid structure itself.’ By contrast, Butler *et al.* [192] didn't find the grid distortion towards rewards that Boccara *et al.* [191] reported, although they did find that rewards affected the arrangement of grid cell firing fields in other ways (grids were translated and rotated, more closely spaced, less elliptical), and also grid cells fired more closer to rewards.

### (c) Grid cells in 3D

**Figure 6.**
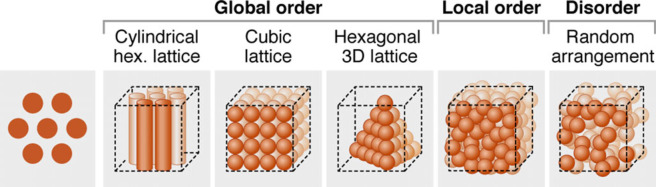
Hypotheses being tested by [11] and [12]. On the left is the 2D hexagonal grid of grid cell firing fields we saw in figure 5. The remaining panels explore potential 3D grid arrangements. Some sort of 3D ‘global order’ was originally hypothesised [193–197]. But Ginosar *et al.* [11] only find evidence of ‘local order’ in bats, whilst the results in rats [12] are consistent with a ‘random arrangement’. From Ginosar *et al.* 2021 [11]. © authors. (Online version in colour.)

Entering our meeting, the great unanswered question was how grid cell firing fields are organized in 3D space? They are regularly ordered on a 2D surface (e.g. the floor of an enclosure) (figure 5), and a number of models suggested that this regular 2D grid was likely a cross-section of a regular 3D grid in 3D space, where each firing field had a fixed angle and distance from one another (‘global order’ in figure 6) [193–197]. As Finkelstein *et al.* [197] noted in 2016: ‘An important future test for this idea would be to record from 3-D grid cells: Do they support the notion of a metric representation of 3-D space?’

It was our privilege to have Gily Ginosar and Kate Jeffery present the two landmark studies on this very question at our meeting; Ginosar on bats flying in 3D space [11], and Jeffery on rats navigating a 3D maze [12]. The surprising answer from both studies is that grid cell firing fields are not arranged in a regular 3D grid, and so have no *global* 3D order [198]. In bats the grid cell firing fields ‘exhibited only local order, creating a locally ordered metric for space’ [11], with firing fields having similar spacing but not similar angles, whilst in rats firing fields were consistent with a random arrangement (no similarity in spacing or angles) [12].

The common finding that grid cell firing fields do not have a global 3D structure is especially striking given that bats and rats diverged evolutionarily 65 million years ago. And it challenges the assumption that grid cells provide a general purpose metric for space:

…suggestions that grid cells are involved in geometric computations … were motivated by the highly geometric, periodic representation of 2D space by grid cells. Given our findings on the absence of global periodicity in 3D, it seems less plausible that 3D grid cells are involved in general purpose geometric computations… (Ginosar *et al.* [11])

Indeed, Grieves *et al.* [12] go further, and suggest that the structure of grid cell firing fields may reflect ‘affordances’ (potential for action). Explaining the difference between their study on rats and Ginosar *et al.* [11]'s study on bats, Grieves *et al.* [12] suggest that it is unlikely to arise from differences between the two species, but instead is likely to be ‘due to how movement patterns through the volumetric spaces can affect grid self-organization.’ For instance, bats could fly in any direction, whilst rats were constrained by the structure of the 3D maze they were navigating. As Kate Jeffery [199] explains, on this account ‘the cognitive map is not fixed and rigid, like an artificial map’, but instead provides ‘a more flexible spatial code in which the map is adapted to the movement possibilities of the space.’ Kate Jeffery [200] continues the discussion in this issue, considering asymmetries and distortions in the firing fields of place cells, grid cells and head-direction cells.

### (d) Cognitive maps and NeuroAI

In another contribution to this volume, Ida Momennejad [201] considers the convergence of neuroscience and machine learning (NeuroAI) (see also [202–205]). Nowhere has this convergence been more apparent than in navigation research. But how should we connect the neural representations (place cells and grid cells) that we are discussing with the computational models of scene understanding (SLAM and reinforcement learning) that we discussed in the computer vision section?

1. SLAM and place cells: One of the earliest models of SLAM (simultaneous localization and mapping) in computer vision was Maja Matarić's ‘Navigating with a rat brain: a neurobiologically inspired model for robot spatial representation’ [206]. Inspired by hippocampal place cells, Matarić aimed for a ‘topological’ rather than Euclidean map of the environment. Like the London tube map, ‘topological’ maps preserve the relationship between landmarks, but not their metric distances or directions. In Matarić's case, her robot could follow the perimeter of its enclosure, but its scene understanding was limited to which landmarks it would encounter in which order. By contrast, later models aimed to provide metric scene representations, with metric information either the explicit input [207] or the implicit output [208, p.107], [209]. Halfway between them, RatSLAM [210–213] aimed for a fine grained topological map that could facilitate shortcuts, but was less than fully metric (‘the map does not follow a strict Cartesian coherence’ [210]).

2. Reinforcement learning and grid cells: By the time grid cells were discovered in 2004–5, the literature's focus had largely shifted to reinforcement learning (learning navigation strategies, rather than hard-coding them; cf. [214]). Reconciling reinforcement learning with place and grid cells has been a key focus of the recent literature (for review see [215]), with both Euclidean and non-Euclidean approaches.

Euclidean: Banino *et al.* [216] and [217] show that grid cell-like patterns can spontaneously emerge when neural networks are trained to perform path integration. Banino *et al.* [216] incorporated this ‘grid network’ into a deep reinforcement learning agent, and further showed that this agent's performance had (unlike a comparison agent) all the hallmarks of a Euclidean spatial metric, such as distance estimation and ‘vector navigation’ (shortcuts to remembered locations [179,218,219]). They therefore conclude that grid cells, in both biological systems and machines, ‘furnish agents with a Euclidean spatial metric’:

…we argue that grid-like representations furnish agents with a Euclidean geometric framework – paralleling the proposed computational role in mammals as an early developing Kantian-like spatial scaffold that serves to organize perceptual experience… (From Banino *et al.* [216].)

Non-Euclidean: By contrast, at our meeting and in [220], Ida Momennejad argues that it's the non-Euclidean aspects of place and grid cells that need accounting for, such as the distortions of grid cell firing fields by rewards, as well as the fact that the majority of distance estimates in the hippocampus reflect path distance (taking into account obstacles) rather than direct Euclidean distance [221,222].

Reinforcement learning has been proposed as a way of capturing these non-Euclidean properties of place and grid cells. Gustafson & Daw [223] argue that an emphasis on path rather than Euclidean distance reflects the fact that place and grid cells ‘are well adapted to support reinforcement learning’, since efficient reinforcement learning requires that the inputs (place and grid cells) are already articulated in terms of the goals of navigation:

Importantly, this exercise views the brain's spatial codes less as a representation for location per se, and instead [as] a *value function* over state space – a mapping of location to value.

More recently, Stachenfeld *et al.* [224,225] argue that place cells are the encoding of ‘successor representations'. In reinforcement learning, ‘successor representations’ [226] provide a model of next steps, affording the flexibility of considering alternatives at each stage (versus model-free reinforcement learning), while avoiding the computational intractability of modelling the whole world (model-based reinforcement learning). On this account, place cells encode the likelihood that a location will be visited given the animal's current navigation strategy, explaining why locations that have the same path distance to a reward can still have different responses from the reward's place cell.

Place cells in the hippocampus have traditionally been viewed as encoding an animal's current location. In contrast, the predictive map theory views these cells as encoding the animal's future locations. (Stachenfeld *et al.* [225])

Momennejad *et al*. [227], Russek *et al.* [228] and Geerts *et al.* [229] also suggest that successor representations capture the semi-flexible navigation strategies of humans and rodents.

Successor representations also promise to invert the traditional relationship between grid cells and place cells. Rather than grid cells being the neutral Euclidean input into place cells [230], Stachenfeld *et al.* [224,225] argue that grid cells are simply a higher-order abstraction (principal component analysis) of the successor representations captured in the place cell cognitive map (see also [231,232]).

### (e) Vision and navigation

At our meeting we intentionally used ‘3D vision’ in a broad (computer vision) sense to include ‘vision for interaction with the 3D world’ (e.g. navigation). But how should we relate the cognitive mapping of space in the hippocampus (place cells) and entorhinal cortex (grid cells) to the perceptual mapping of space in the visual cortex? Can navigation affect vision?

At our meeting Aman Saleem discussed his and colleagues' findings in [233] that the location of rewards alters firing rates in the mouse primary visual cortex as well as the hippocampus, suggesting an influence of cognitive maps all the way down to the earliest retinal maps in the cortex. Saleem *et al.* [233] therefore conclude that:

…visual responses in V1 [primary visual cortex] are controlled by navigational signals, which are coherent with those encoded in hippocampus … The presence of such navigational signals as early as a primary sensory area suggests that they permeate sensory processing in the cortex.

The effect of self-motion on vision in mice and humans is further explored by Aman Saleem and colleagues (Horrocks, Mareschal & Saleem [234]) in this issue.

## 3. Human vision

Like computer vision and animal navigation, competing interpretations of human 3D vision alternate between adopting no 3D model, a 3D model that recovers metric scene properties, or a 3D model that doesn't recover metric scene properties. These different approaches are summarized in table 2.

**Table 2.** Models of human 3D vision.

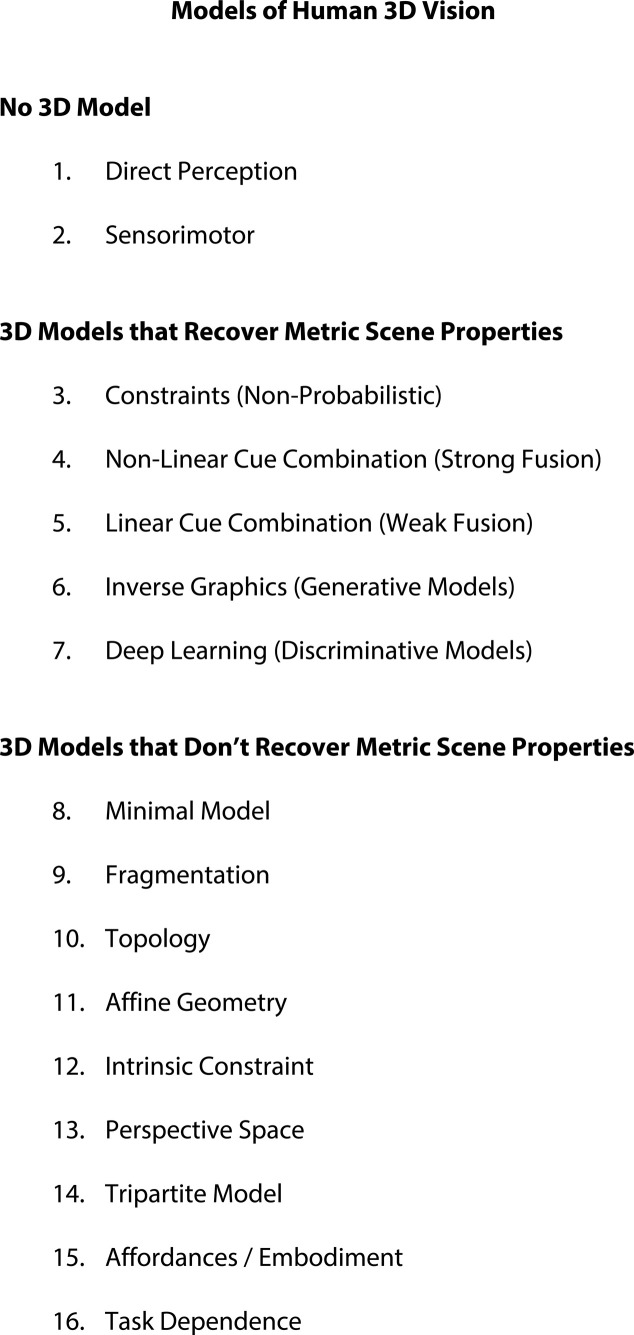

### (a) No 3D model

1. Direct Perception: The most famous ‘no model’ account of human vision is James Gibson's ‘direct perception’ [144,235,236], according to which we directly perceive the structure of the world through ‘invariants’ in the constantly changing retinal image:

…*perceiving* is a registering of certain definite dimensions of invariance in the stimulus flux … The invariants are invariants of structure [236, p.249].

The theory of the extracting of invariants by a visual system takes the place of theories of ‘constancy’ in perception, that is, explanations of how an observer might perceive the true colour, size, shape, motion, and direction-from-here of objects despite the wildly fluctuating sensory impressions on which the perceptions are based. [236, p.311].

But Gibson's proposal has been given a variety of interpretations by the contemporary literature. Wagner and colleagues [237] suggest that ‘Gibson's … doctrine of realism implies that visual space should be strictly Euclidean … ’ Warren [238] advances an ‘affine’ interpretation: ‘humans do not in fact recover Euclidean structure—rather, they reliably perceive qualitative shape (hills, dales, courses and ridges), which is specified by the second-order differential structure of images.’ Finally, Tsao & Tsao [239] argue for a ‘topological’ approach.

2. Sensorimotor: Sensorimotor (pixel-to-action) accounts of human vision have also been influential [240]. We saw sensorimotor (pixel-to-action) accounts previously in computer vision with Sergey Levine's work on reinforcement learning. In this issue Andrew Glennerster [241] argues that model-free reinforcement learning (without a 3D model) is a good model for human 3D vision, and his paper aims to show ‘how a policy network could support the same behaviour as a system that uses a 3D reconstruction of the scene.’

### (b) 3D models that recover metric scene properties

3. Constraints (Non-Probabilistic): The dominant approach to human and computer vision in the 1970s–80s was specifying the physical constraints on how the retinal image was produced, so that the inverse optics question (what 3D scene produced this 2D image?) had a unique solution: ‘the resulting operation is defined uniquely by constraints it has to satisfy’ (Marr [17, p.23]). However, in the late 1980s and early 1990s, it became apparent that simply specifying constraints would not suffice, although some argue that we simply have too limited a notion of these constraints [242,243].

4–6 (Below). Bayesian approaches: Instead, the visual system would have to decide which of the remaining potential percepts were more or less likely, framing perception as a probabilistic process: ‘The principle aspect of this approach is the probabilistic representation of constraints.’ (Clark & Yuille [244, p.218]). Bayesian models have been the dominant approach to human 3D vision for the past 25 years [245–248], and can be articulated in one of three ways:

4. Linear cue combination (weak fusion): The leading approach to human 3D vision [246,249] treats 3D vision simply as a problem of eradicating of sensory noise. It breaks 3D vision down into a series of ‘cues’ (e.g. stereo vision, motion parallax, structure from motion, perspective, shading), and assumes each cue gives an accurate (undistorted, unbiased) but imprecise (vague, noisy) depth estimate. It then reduces the effect of sensory noise by taking a weighted average of the individual cues: the less noisy a cue is, the more weight its estimate is given. Empirical support can be found in [250–261].

However, there are two key concerns with this approach. First, it assumes each cue gives an unbiased (undistorted) depth estimate. But we will see below that this isn't the case. And Domini & Caudek [262] argue that if ‘the estimates of the world properties are biased, … it is meaningless to maximize reliability’ (cf. [260]). Second, a significant number of studies are inconsistent with linear cue combination's prediction that the less noisy a cue is, the more weight its estimate is given [263–268] (see also [269] and [270]'s related methodological concerns).

**Figure 7.**
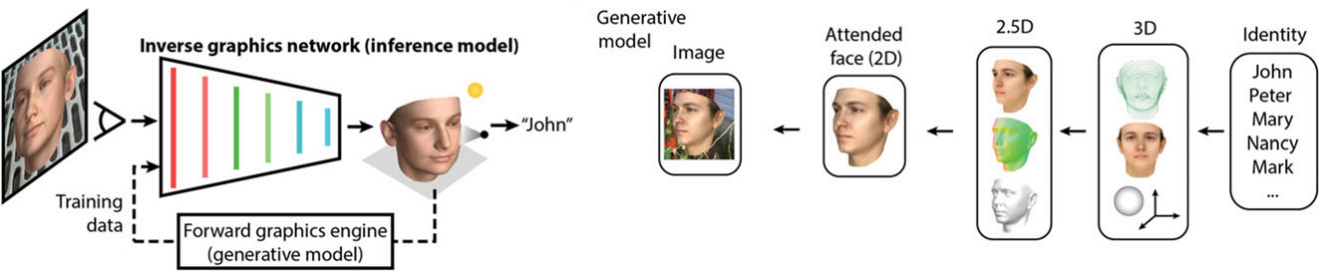
Inverse graphics network from [281]. How do we recognize someone as ‘John’? Rather than train a neural network to directly identify people (2D image → identity), [281] use a ‘generative model’ (on the right) (identity → 3D scene → 2D image) to produce training data for an ‘inference model’ (on the left) that first estimates the 3D scene properties of the 2D image (2D image → 3D scene), before using this 3D scene estimate to identify the person (3D scene → identity). © authors. (Online version in colour.)

5. Nonlinear cue combination (strong fusion): By contrast, nonlinear cue combination assumes some degree of bias in the individual cues, and is really about ‘the constraints needed to solve sensory information processing tasks, rather than just a method for reducing the effects of sensor noise.’ (Clark & Yuille [244, p.222]). The focus is still on metric scene recovery, so that the cue combination rules ‘inverting the world-image mapping are sufficient and, most importantly, *valid*.’ (Clark & Yuille [244, p.222]). Nonlinear cue combination was originally more popular in the early 1990s, and ranges from the addition (rather than averaging) of cues [271,272], through to highly sophisticated interdependencies between cues [244,273].

6. Inverse graphics (generative models): Other, more recent, Bayesian accounts have done away with ‘cues’ altogether, and ask: ‘what arrangement of lights, surfaces, and materials would give rise to this specific 2D image?’ [274–280]. This ‘inverse graphics’ approach relies on ‘analysis by synthesis': simulating 2D images of different 3D scenes to see under which 3D scene configuration the actual 2D image is most likely. Yildirim *et al.* [281] (figure 7) divide this into two stages, where a ‘synthesis’ model (‘generative model’, on the right in figure 7) simulates 2D images of 3D scenes, which are then used to train a separate ‘analysis model’ (‘inverse graphics network’, on the left in figure 7).

7. Deep learning (discriminative models): In contrast to ‘inverse optics’ models (3–6 in table 2), deep learning models can be thought of as reflecting a ‘statistical appearance model’ [282–284]: ‘rather than learning the mappings between image quantities (cues) and physical quantities, we learn to represent the dimensions of variations within and among natural images, which in turn arise from the systematic effects that distal properties have on the image.’ (Fleming & Storrs [284]). See also the discussion of the ‘generative’ versus ‘discriminative’ approaches in [285]. Strictly speaking, this approach aims to capture a compact representation of the variables that determine image structure (latent variables), rather than a representation of physical scene structure itself. However, as Fleming & Storrs [284] suggest, one may lead to the other: ‘we may end up with internal representations that are well suited for describing the *distal* scene factors that have created those images.’

### (c) Failures of metric scene reconstruction

Models 3–7 in table 2 provide different ‘normative’ or ‘ideal observer’ models [286] of how 3D vision ‘ought’ to act if it were trying to estimate the metric properties of the scene. However, there's two ways in which human 3D vision regularly fails to live up to this standard of metric scene reconstruction.

**Figure 8.**
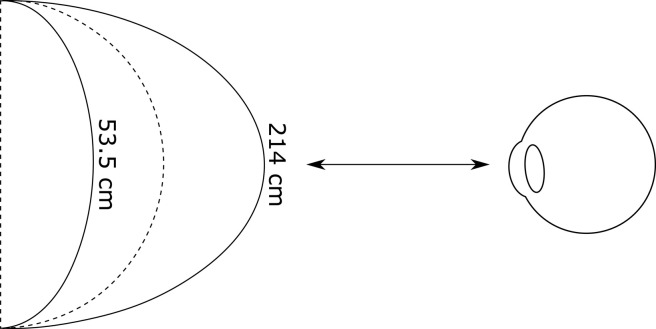
Schematic of the task in [287]. Participants set the depth of the cylinder so that its depth appeared to be proportional to its height (the dotted line). At near viewing distances (53.5 cm) the cylinder they produced was compressed in depth, while at far viewing distances (214 cm) the cylinder they produced was elongated in depth.

1. Constancy (distortions): First, our depth perception is subject to considerable distortions. One vivid illustration is stereo vision (depth from disparity). In Johnston [287], participants viewed a cylinder whose depth was defined by stereo vision (disparity) alone. Their task was simple. Set the cylinder's depth so it was proportional to its height (the dotted line in figure 8). But the cylinder produced varied drastically in depth with the viewing distance. At 53.5 cm the cylinder they produced was compressed in depth, suggesting that depth from disparity is accentuated at close distances, while the cylinder they produced at 214 cm was elongated in depth, suggesting that depth from disparity is compressed at far distances. And these 3D shape distortions persist even in the presence of other cues [288,289].

Similarly, we seem to experience less depth when we look at the world with one eye closed, but there's little justification for why this should be the case if vision is estimating metric scene properties using the available information [290,291, pp.13–16].

Domini & Caudek [262] rightly consider ‘the large failures of constancy of 3D metric structure over changes in viewing distance and/or orientation to be an important empirical finding that needs to be addressed by any theory’. First, metric cue integration accounts try to suggest that ‘failures to observe depth constancy may be due to the influence of unmodelled flatness cues such as blur and accommodation’ [249]. But this doesn't seem to fully meet the challenge. Second, others try to suggest that these distortions merely affect our visual experience of depth (qualia), but not our metric scene estimates [291, pp.13–16]. But our visual experience of depth is exactly what a theory of 3D vision should explain. Third, some try to suggest that these distortions are a way of the visual system conveying how reliable our metric scene estimates are [292]. But this is not supported by the data [293].

2. Consistency (conflicts): Second, our judgements about visual space are often marked by pervasive inconsistencies. Di Luca *et al*. [294] find inconsistencies between estimates of depth, slant and curvature. Koenderink [295] finds inconsistencies between global and local depth judgements. Loomis *et al*. [296–298] find inconsistencies between absolute distance judgements and relative depth judgements. Koenderink *et al*. [299] find conflicting judgements of fronto-parallels. And Svarverud *et al.* [300] find visual space can be ‘broken’, with no consistent ordering between objects. Illusions also provide insights into inconsistency: Gillam & Chambers [301] find that position and size are inconsistent in the Müller-Lyer illusion, while Smeets *et al.* [302] find that the perceived centre of the Judd and Poggendorff illusions depends on the order in which the points are constructed. There's also a debate over whether we experience conflicting shape percepts when we see a rotated coin both as a circle and as an ellipse (Morales *et al*. [303–305] vs Linton [306] vs Burge & Burge [307]).

### (d) 3D Models that don't recover metric scene properties

On ‘normative’ or ‘ideal observer’ models, these failures of metric scene reconstruction reflect a failure of evolution to live up to our rational standards of what vision ‘ought’ to be doing (Landy *et al.* [249]):

Of course, there remains the possibility that we have characterized the sensory information *and the task* correctly, but the nervous system simply has not developed the mechanisms for performing optimally (Landy *et al.* [249], emphasis added)

By contrast, for models 8–16 in table 2, these failures of metric scene reconstruction suggest that we haven't specified the task correctly. Instead, these failures give us an important insight into the very different task that the visual system has set itself.

So a number of authors, including three of the present authors (Linton, Vishwanath, Domini), see the failures of metric scene reconstruction as a reason to question whether human 3D vision is trying to extract the metric 3D properties of the environment in the first place. Vishwanath [308] argues that 3D vision is ‘*the presentation* of causally efficacious visual information rather than an *inference* to objective external reality.’ Domini & Caudek [262] argue that ‘the goal of the visual system is to guarantee a successful interaction between the observer and the environment without recovering metric 3D (3D) information’. And Linton [309], p. 74 argues that 3D vision ‘operates purely at the level of *phenomenal geometry*, and makes no claims about the *physical geometry of the physical world’*.

But if human 3D vision isn't trying to estimate the metric properties of the environment, what else could it be doing? The following models provide nine distinct alternatives.

8. Minimal model: In this issue, Paul Linton [310] argues for a ‘minimal model’ of 3D vision.

First, in order to answer the challenge of ‘inconstancy’, Linton decouples stereo vision from estimating scene properties such as distance and shape, rejecting ‘triangulation’-based accounts of stereo vision that date back to Kepler [311] and Descartes [312]. Instead, on his account, stereo depth is simply a solution to a different (and entirely internal) problem, the eradication of rivalry between the two retinal images. Indeed, rather than the resulting ‘inconstancy’ being a problem to be solved, Linton argues that it's primarily through this ‘inconstancy’ that we judge size and distance.

Second, in order to answer the challenge of ‘inconsistency’, Linton argues that stereo vision and non-stereo cues (and therefore the inconsistencies between stereo vision and non-stereo cues) operate at different levels, with stereo vision affecting our perception (visual experience) of depth, whilst non-stereo cues (such as motion, perspective and shading) merely affect our cognition (judgements) of depth.

9–13 (Below). Qualitative models of scene geometry: The following five approaches suggest that human 3D vision captures scene geometry in a qualitative (fragmentary and/or distorted) sense, but differ as to the exact way in which scene geometry is fragmented and/or distorted. Often this takes the form of asking how loose (or permissive) the mathematical transformation from physical space to visual space is? From more to less permissive: Topology → Projective Geometry (Perspective) → Affine Geometry → Euclidean Geometry.

9. Fragmentation: A common response to conflicting depth estimates of the same 3D scene is to say that humans are only capable of local, and often inconsistent, depth judgements. For instance, Koenderink [295] suggests:

Observers are quite content to live with any number of mutually inconsistent fragmentary representations since they can blindly depend on the consistency of the physical world.

And illusions lead [301,313] and [302] to reach a similar conclusion. Asking ‘Does visual space exist?’, Smeets *et al.* [302] conclude:

It might be more fruitful to abandon the concept of a geometrically consistent perceptual (or motor) space altogether. Instead, one can regard perception as a set of independent local estimates of various spatial attributes.

However, Koenderink *et al.* [314] raise the possibility that while depth judgements are fragmentary, they're still consistent, arguing that you can build up a coherent global map from observers' local depth judgements. But Koenderink *et al.* [314] argue that observers are unable to build up a coherent global map for themselves since they experience visual space neither globally nor locally, but as a patchwork of ‘hills’ and ‘troughs’:

Possibly the data structure itself is not a whole, but rather a quilt of locally coherent, but mutually only weakly synchronized patches.

10. Topology: Nowhere has the shift in thinking about human 3D vision from a quantitative ‘inverse optics’ approach to a qualitative approach been more apparent than in ‘shape-from-shading’. Initial research in the 1980s tied human vision closely to computer vision [315]. Now leading figures from that tradition seek to sharply distinguish human vision from ‘inverse optics’. So, Jan Koenderink, Andrea van Doorn, and colleagues suggest [316]:

It may well be the case that the whole notion of shape from shading is spurious … , and that biological vision research should leave it to computer vision engineers.

Similarly, Steven Zucker, in ‘On qualitative shape inferences: a journey from geometry to topology’ (2020) [317] argues:

…we argue that the perception of shape is qualitative, not quantitative, a point that has been well understood in visual psychophysics for decades. This suggests that we should not be seeking to solve the shape-from-shading equations, but should look for qualitative (that is, topological) solutions instead.

And both Koenderink *et al*. and Zucker point to the considerable differences not just between observers' 3D estimates and reality, but also between observers themselves.

For Kunsberg & Zucker [318,319], this qualitative approach is best expressed through ‘topology’, the study of what remains true about a surface after it has been distorted in an arbitrary way. For instance, if we think about deforming a mesh, neighbouring points on the mesh before deformation will still be neighbouring points afterwards. For Zucker, the topology of the surface that emerges in shape from shading is anchored in a few places by ‘critical contours’ (such as peaks and troughs), which form ‘a kind of scaffold on which the shape can be readily built.’ But beyond that, each person's interpretation of the surface is largely subjective and unconstrained.

11. Affine geometry: By contrast, Koenderink *et al*. [320] suggest observers can recover something more than topology, but still less than Euclidean geometry, noting that observers are ‘surprisingly close to the physical layout modulo a gauge transformation, with the deviations being mainly an isotropic rotation and scaling.’ This is ‘affine geometry’, which captures the Euclidean geometry subject to a homogeneous stretch or shear. Affine geometry is more structured than topology, preserving the relationship between parallel lines, but not the distance or angles between points.

As we saw earlier in computer vision, the information from both stereo vision (disparity) and structure from motion also lends itself to analysis at the affine level. But evidence of whether humans can recover affine structure from stereo vision or motion is equivocal. Domini *et al.* [321] find that structure from motion is not affine, while [322,323] find that structure from motion is affine but stereo vision is not. Todd *et al.* [324] did however find that whilst the mapping from physical space to visual space is not affine for stereo vision, stereo vision is at least internally consistent in an affine sense (‘perceptions had an internally consistent affine structure’), since line bisections in different directions were consistent with one another.

More broadly, Wagner *et al.* [325] suggest that size and distance judgements are best thought of as affine transformations of physical space, and Glennerster *et al.* [326] that pointing errors in virtual spaces reflect affine distortions. Affine geometry also played a key role in early versions of Domini & Caudek's ‘intrinsic constraint’ cue combination model [262,327]: ‘Our main claim … is that the brain extracts from retinal signals the local affine information of environmental objects.’

12. Intrinsic constraint: In this issue Fulvio Domini [328] presents a new version of the ‘intrinsic constraint’ model. On this new account, perceived depth is still linearly related to (and therefore still an affine transformation of) physical depth. But what this new ‘intrinsic constraint’ model is trying to do is not estimate affine depth *per se*, but simply maximize the 3D signal in the image (while minimizing nuisance variables, such as viewing conditions and materials). To achieve this, the ‘intrinsic constraint’ model uses a ‘vector sum’ that adds (rather than averages) the depth estimates from the individual cues, so that the more cues you have (and the more 3D signal is in the image), the more depth you see. While this model aims to achieve a more stable representation across viewing conditions, it also explains why adding or removing cues can lead to inconsistent depth estimates.

13. Perspective space: Train tracks appear to converge as they recede in distance, suggesting that instead of an affine transform (which preserves parallel lines), visual space is a perspective projection of Euclidean space (with visual space converging to a vanishing point) [329–333]. Wagner *et al.* [237] find that human judgements correspond better to a perspective projection than an affine transform. But what's so surprising about the perspective space account is just how shallow visual space appears to be. Erkelens [332] asked participants to match the perceived convergence of railway lines using compasses, and the vanishing point inferred was no further than 6 m, suggesting that visual space is compressed in depth, like a bas relief, to fit within 0–6 m of physical space.

14. Tripartite model: Perhaps we should think of visual spaces, rather than one single visual space. In this issue Dhanraj Vishwanath [334] argues for a ‘tripartite model’ of visual space, according to which there are three ‘distinct and dissociated encodings' for (a) 3D shape, (b) ‘egocentric’ (observer to object) distances, and (c) ‘exocentric’ (object to object) distances. However, often viewing conditions will only support one or two of these encodings, explaining the ‘inconsistencies’ reported in the literature above.

Vishwanath argues for these three distinct encodings based on their different experiential ‘qualities’, in contrast to standard models that ‘typically do not make a fundamental distinction among these different modes of spatial experience’. For instance, the dual nature of pictures reflects the fact that pictorial space supports 3D shape perception but not ‘egocentric’ and ‘exocentric’ distance perception, whilst the vivid separation in depth typically associated with stereo vision (depth from disparity) reflects the ‘exocentric’ distance encoding.

15. Affordances/Embodiment: The past couple of decades have seen the rise of a ‘pragmatic turn’ in cognitive science, according to which ‘cognition should not be understood as providing models of the world, but as subserving action’ [335–337]. This has led to increased interest in the visual processing of ‘affordances’ (potential for action) [236,338]. We have already encountered one such affordance (traversability) in our discussions of robot navigation [104] and rats navigating 3D mazes [12]. And Sarah Creem-Regehr's [339] talk at our meeting focused on affordances in virtual and augmented reality.

But what are the implications for 3D vision? Do affordances replace our perception of 3D surfaces, as Sergey Levine [340] suggests they ought to for robotics? In some passages Gibson suggests so (‘What animals need to perceive is not layout as such but the affordances of the layout’, Gibson [341, pp.157–158]), but contemporary Gibsonians reject this (Warren [342]).

Instead, the closest we come to this view are ‘embodied’ theories of perception [343,344] that claim that ‘explicit awareness of spatial layout varies not only with relevant optical and ocular-motor variables but also as a function of the costs associated with performing intended actions.’ The classic claim is that hills are perceived as steeper when you wear a heavy backpack or if you are elderly ([345]; see also [346–348]). However, this theory has been criticized from both experimental [349] and theoretical [350,351] perspectives, with the suggestion being that the effect of the rucksack is a consequence of participants' trying to ‘act as they should’ in the experiment. For the latest iteration of this debate see [352–355].

16. Task dependence: Still, perhaps there is something to the idea of tying scene estimates to the task being performed. Indeed, the suggestion that 3D vision is task-dependent is one of the most common responses to the ‘inconsistencies’ in 3D vision outlined above. For example, Glennerster *et al.* [356] found that depth constancy for stereo vision (disparity) depends on the nature of the task, and the same was found for tasks involving both stereo vision and motion parallax [357]. Norman *et al*. [358] tested the relationship between objects in action space and found that: ‘Whether a Euclidean or affine compressed visual space was obtained depended not upon any characteristic of the visual stimuli, but upon the specific task employed by the observer.’ Wagner & Gambino [325] ‘embrace the idea that visual space is a living, malleable entity whose geometry changes with experimental conditions and shifts in observer attitude.’ Finally, Warren [338] concludes that ‘there *is* no consistent visual space. Rather, perception by an active agent is task-specific and information-driven, such that judgements of different properties of layout are based on different optical variables.’

But task dependence doesn't necessarily mean giving up on the concept of visual space. Mel Goodale and David Milner have long argued that task-dependence implies two models of visual space, one for conscious visual perception and the other for action [359–361]. A key claim of this account is that vision for perception is distorted by illusions, whilst vision for action is not. But an alternative explanation for this effect is that eye movements are different in perception and action, explaining the different effect of illusions. So, in this issue, to rule this out, Whitwell *et al.* [362] show that the effect still persists even when there are no significant differences between eye movements in perception and action.

### (e) Virtual reality

Virtual reality enables us to study perception in a more ecologically valid way that is closer to the real world than conventional displays. But it also enables us to test vision in a more ecologically invalid way by decoupling the visual and physical consequences of our actions. This technique is used in two papers in this issue. First, Horrocks, Mareschal, & Saleem review [363] how virtual reality is being used to study the effects of locomotion on optic flow in humans and mice. Second, Maselli, Ofek, Cohn, Hinckley & Gonzalez-Franco [364] test how participants respond to displacing the location of a virtual (seen) hand relative to their physical (unseen) hand as they reach for an object, and find more efficient corrections towards the body midline.

However, we are still in the process of understanding the limitations of virtual reality. At our meeting, Douglas Lanman outlined the progress that his Display Systems Research team at Meta (Facebook) Reality Labs is making towards the ‘visual Turing test’: creating a display indistinguishable from reality [365,366]. But he also cautioned against equating virtual reality with real-world vision given the optical distortions that exist in virtual reality displays [367–369]. The concern that virtual reality doesn't reflect real-world perception is also shared by two papers in this issue. First, Creem-Regehr, Stefanucci & Bodenheimer [339] show how distances are underestimated in virtual reality, and the strategies that can be used to improve distance perception. Second, Rzepka, Hussey, Maltz, Babin, Wilcox & Culham [370] find that participants rely far more on the familiar size of objects when making distance judgements in virtual reality than they do in the real world.

### (f) 3D space and visual impairment

The final two contributions to this issue explore how visual impairments affect our perception of space, and the ability of the human brain to adapt to these impairments.

First, 8% of the general population appear to have no stereo vision (they are unable to extract depth from disparity) [371,372]. Poor stereo vision is known to affect fine motor skills (such as threading a bead on wire) [373], reaching and grasping [374], and walking across uneven terrain [375]. Sue Barry [376] (‘Stereo Sue' in Oliver Sacks’ *The Mind's Eye* [377]) and Bruce Bridgeman [378] provide vivid personal descriptions of how recovering stereo vision transformed their visual experience:

Gaining stereovision, I thought, would augment my perception of depth but not change it in any fundamental way. So, I was completely unprepared for my new appreciation of space… (Barry [376], p.111)

Extrapolated to the world population, stereo vision deficits affect over half a billion people, so a key concern is better understanding what causes stereo vision deficits, how they affect our interactions with the world, and how they might be treated. In this issue Niechwiej-Szwedo, Colpa & Wong [379] review the effect that amblyopia (lazy eye) has on the development of reaching and grasping, documenting how young children lag behind their peers, and older children develop compensatory strategies.

Second, recovery from early blindness can cause very selective visual deficits. As Ione Fine and colleagues' found with Mike May when he recovered his sight, his perception of visual motion was relatively ‘normal’, whilst his perception of 3D form remained permanently impaired [380,381]. In this issue, Fine & Park explain this by pointing to the fact that early blind individuals use auditory motion for many of the tasks we would typically attribute to 3D vision (e.g. navigating a busy interaction using noise from passing cars). But how is this possible? Fine & Park [382] find that this ability relies on the auditory system adopting brain area hMT+, that's associated with visual motion processing in normal observers, but it changes the nature of the motion processing that hMT+ engages in to accommodate the low spatial resolution of auditory information.

## 4. Conclusion

As we noted at the beginning, the purpose of our meeting was to capitalize on a brief moment when computer vision, animal navigation, and human vision are all pausing and asking what the most appropriate representation for 3D vision and action really is? The argument of this article, and indeed of this issue, is that our understanding of how brains and computers do 3D vision is at a crossroads. As Andrew Glennerster [383], one of the contributors to this issue, notes, ‘we're moving away from the idea that what the brain does is something complicated, which is easy for us to understand’, namely a metric 3D map of the environment, ‘toward the view that the brain does something which is easy for it to do, but really quite hard for us to understand’. This issue presents sixteen perspectives on what that ‘something’ might be. But it's meant to be the beginning of a conversation, not the end. And, at a time when neuroscience, and science in general, is thought to be in the midst of a ‘theory crisis’^2^, our hope is to have put these theoretical questions back at the centre of 3D vision.

## Data Availability

This article has no additional data.

## Endnotes

1 Office of Naval Research grant ONR MURI N00014-16-1-2007. Multidisciplinary University Research Initiative (MURI): ‘The goal of this MURI team is to develop machines that have the following capabilities: … Achieve deep understanding of scenes and events through joint parsing and cognitive reasoning about appearance, geometry, functions, physics, causality, intents and belief of agents’. Members (USA): Song-Chun Zhu, Martial Hebert, Abhinav Gupta, Nancy Kanwisher, Josh Tenenbaum, Derek Hoiem, Brian Scholl, Tao Gao, Noah D. Goodman. (UK): Philip Torr, Philippe Schyns, Andrew Glennerster, Ales Leonardis.

2 See [384, 39:33–41:40], [385–387], [388, 3:05–4:45], [389–398].
